# Electric Field Application *In Vivo* Regulates Neural Precursor Cell Behavior in the Adult Mammalian Forebrain

**DOI:** 10.1523/ENEURO.0273-20.2020

**Published:** 2020-08-21

**Authors:** Elana Sefton, Stephanie N. Iwasa, Taylor Morrison, Hani E. Naguib, Milos R. Popovic, Cindi M. Morshead

**Affiliations:** 1 Institute of Biomedical Engineering, University of Toronto, Toronto, Ontario, Canada, M5S 3G9; 2 The KITE Research Institute, Toronto Rehabilitation Institute - University Health Network, Toronto, Ontario, Canada, M5G 2A2; 3 CRANIA, University Health Network and University of Toronto, Toronto, Ontario, Canada, M5G 2A2; 4 Department of Mechanical & Industrial Engineering, University of Toronto, Toronto, Ontario, Canada, M5S 3G8; 5 Department of Materials Science & Engineering, University of Toronto, Toronto, Ontario, Canada, M5S 1A1; 6 Department of Surgery, University of Toronto, Toronto, Ontario, Canada, M5T 1P5

**Keywords:** cell survival, EGFR, electrical stimulation, neural stem cells, quiescence, sFRP2

## Abstract

Deep brain stimulation (DBS), which uses electrical stimulation, is a well-established neurosurgical technique used to treat neurologic disorders. Despite its broad therapeutic use, the effects of electrical stimulation on brain cells is not fully understood. Here, we examine the effects of electrical stimulation on neural stem and progenitor cells (collectively neural precursor cells; NPCs) from the subventricular zone in the adult forebrain of C57BL/6J mice. Previous work has demonstrated that adult-derived NPCs are electro sensitive and undergo rapid and directed migration in response to application of clinically relevant electric fields (EFs). We examine NPC proliferation kinetics and their differentiation profile following EF application using *in vitro* and *in vivo* assays. *In vitro* direct current electrical stimulation of 250 mV/mm is sufficient to elicit a 2-fold increase in the neural stem cell pool and increases neurogenesis and oligogenesis. *In vivo,* asymmetric biphasic electrical stimulation similarly increases the size of the NPC pool and alters neurogenesis. These findings provide insight into the effects of electrical stimulation on NPCs and suggest its potential use as a regenerative approach to neural repair.

## Significance Statement

Electrical stimulation promotes neural precursor cell (NPC) migration. In this study, we demonstrate that electrical stimulation, in addition to cell migration, can also expand the size of the NPC pool and enhance neurogenesis, both *in vitro* and *in vivo*. Using electrical stimulation to activate neural stem cells could be a powerful tool to promote tissue repair.

## Introduction

Neural stem and progenitor cells, collectively referred to as neural precursor cells (NPCs), are found within the periventricular region of the adult brain (Reynolds and Weiss, 1992; Weiss et al., 1996) in well-defined niches comprised of numerous cells and factors that provide cues regulating their behavior (Obernier and Alvarez-Buylla, 2019; Ruddy et al., 2019). Environmental signals that modulate NPC behavior, such as those that alter cell cycle kinetics, cellular migration, and/or differentiation into mature neural cell phenotypes, prove useful for neurorepair strategies (Minnerup et al., 2011; Dadwal et al., 2015; Nusrat et al., 2018; Rogall et al., 2018; for review, see Hermann et al., 2014).

Electric fields (EFs) are a critical environmental signal present during development and in adulthood. During development, EFs play a role in morphogenesis and tissue formation whereby disrupting endogenous EFs leads to severe defects and aberrant neural development (Hotary and Robinson, 1992; Metcalf and Borgens, 1994). In the adult, EFs generated following injury are critical for proper wound repair and regulate cell behavior (McCaig et al., 2005; Iwasa et al., 2017; McLaughlin and Levin, 2018). Deep brain stimulation (DBS) involves the clinical application of exogenous EFs to treat diseases and disorders such as essential tremors, Parkinson’s disease (PD), dystonia, pain, major depressive disorders, and other intractable disorders (Deuschl et al., 2006, Kringelbach et al., 2007; Chiken and Nambu, 2015). Current evidence supports the ability of applied EFs to enhance neuroplasticity through modifying inhibitory and excitatory activation of neurons (Benabid, 2003; Kringelbach et al., 2007; Chiken and Nambu, 2015; Jakobs et al., 2019); activating resident astrocytes leading to changes in calcium and glutamate (Fenoy et al., 2014), and increasing the release of brain-derived neurotrophic factor (BDNF; Cho et al., 2013). With respect to NPCs, Stone et al. (2011) found an increase in cell proliferation in the dentate gyrus, a neurogenic region of the adult brain following DBS, and Vedam-Mai et al. (2014) showed an increase in proliferating NPCs in the brains of PD patients that underwent electrical stimulation. These studies highlight the potential for electrical stimulation to promote neuroplasticity.

NPCs are electrosensitive cells. *In vitro*, NPCs undergo rapid and directed migration toward the cathodal direction of an externally applied EF (Babona-Pilipos et al., 2011, 2015; Iwasa et al., 2017). *In vivo*, endogenous EFs exist within the brain parenchyma (Cao et al., 2013; Iwasa et al., 2019) and have been shown to promote NPC migration to the olfactory bulb. Further, application of external EFs can alter endogenous neuroblast migration (Cao et al., 2013) and guide migration of transplanted NPCs in rodents (Feng et al., 2017; Iwasa et al., 2019). However, few studies have examined the direct effects of EFs on NPC kinetics (Ariza et al., 2010; Chang et al., 2011). Herein, we examine the effects of electrical stimulation on NPC behavior in the periventricular niche of the adult forebrain.

We show that acute *in vitro* and *in vivo* electrical stimulation is sufficient to elicit a 2-fold increase in the size of the NPC pool, as measured by the number of clonally derived neurospheres, through enhanced survival. *In vitro*, neurospheres from cultures exposed to EFs were more neurogenic compared with unstimulated cultures. *In vivo*, EF application resulted in a concomitant increase in NPC proliferation and neurogenesis. Together, these findings demonstrate that electrical stimulation modulates NPC behavior, which may have potential in the therapeutic application of this technique.

## Materials and Methods

### Animals

All animal work was approved by the University of Toronto Animal Care Committee in accordance with institutional guidelines (protocol no. 20011279) and with the federally mandated standards (Canadian Council of Animal Care), provincial legislation (Animals for Research Act, R.S.O. 19990, c.A.22) and the Local Animal Care Committee. Surgeries were performed on 7- to 11-week-old C57BL/6J male mice (027, Charles River) or heterozygous GFAP::GFP mice (003257, The Jackson Laboratory) which overexpress green fluorescent protein (GFP) under the control of the glial fibrillary acidic protein (GFAP) promoter (Zhuo et al., 1997).

### Neurosphere assay

NPCs were isolated from the periventricular zone of the adult mouse as previously described (Morshead et al., 2002; Babona-Pilipos et al., 2011, 2012). Briefly, mice were anesthetized with isoflurane and cervically dislocated. The brains were quickly removed and the periventricular tissue was microdissected. Tissue was enzymatically dissociated in hyaluronidase (1157 units/ml, Millipore-Sigma), trypsin (1.33 mg/ml, Millipore-Sigma), and kynurenic acid (0.13 mg/ml, Millipore-Sigma) for 25 min at 37°C and mechanically dissociated through trituration. The solution was spun down and resuspended in trypsin inhibitor (0.33 mg/ml, Worthington Biochemical Corporation). The suspension was washed with serum-free media (SFM; 1 × DMEM/F12, 0.6% glucose, 0.1% NaHCO_3_, 5 mm HEPES buffer, glutamine, defined hormone and salt mixture, and penicillin/streptavidin). The cells were plated in SFM with epidermal growth factor (20 ng/ml; Millipore-Sigma), basic fibroblast growth factor (20 ng/ml; Millipore-Sigma), and heparin (2 μg/ml, Millipore Sigma; herein referred to as supplemented SFM) in chambers for *in vitro* stimulation (see section on Chamber preparation for *in vitro* stimulation), or in 24-well plates at 5000 cells/μl that were cultured for 7 d (in neurosphere conditions) for assessment of the size of the NPC pool following *in vivo* stimulation. When plated at clonal density (10 cells/µl), the number of spheres with diameters >80 µm were counted as neurospheres, and the number of spheres with diameters 50–80 µm were counted as progenitor colonies (Coles-Takabe et al., 2008).

### Chamber preparation for *in vitro* stimulation

Chambers were modified based on previously published designs (Babona-Pilipos et al., 2012). Briefly, square glass cover slides (no. 1; 22 × 22 × 0.17 mm; VWR) and 60 × 15 mm Petri dishes were exposed to UV light overnight and cover slides were then sealed to the base of the Petri dishes (VWR) using silicone vacuum grease (VWR). Plates were sterilized with 70% ethanol (5 min), followed by 3 × 5 min washes with sterile ddH_2_O; 2 min before cell seeding, 50 μl of 1:1 mixture of hyaluronan/methylcellulose (HAMC) prepared in SFM (Ballios et al., 2015) was placed in the center of the chamber. HAMC hydrogel was used to promote cell viability and keep the cells in the center of the chamber (Ballios et al., 2015; Ho et al., 2019). After cell seeding (see section on In vitro stimulation), 925 μl of supplemented media was added to the chamber; the viscosity of the gel ensured the cells remained in the center of the chamber.

### *In vitro* stimulation

Once isolated, cells were resuspended in 100 μl of supplemented SFM (two chambers, 50 μl per chamber) for a final cell density of 5000 cells/μl. A total of 50 μl of cell suspension was added to each chamber. Grease strips 5–7 mm high were placed on either side of cell suspension to create a trough, and 925 μl of supplemented SFM was added for a final volume of 1 ml in the central trough. The chamber was transferred onto the stage of a temperature-controlled, CO_2_-controlled, and humidity-controlled Zeiss Observer Z1 microscope (Zeiss). Two 15-cm-long pieces of PVC tubing (2.38 mm i.d., 3.97 mm o.d.; Fisher Scientific) were filled with 1.5% (w/v) agarose gel. Two 60 × 15 mm Petri dishes were placed on the stage, one on either side of the stimulation chamber, and filled with 7.5-ml SFM. Two Ag/AgCl electrodes (Alfa Aesar) were placed into the peripheral Petri dishes, and all three dishes were bridged with the agarose gel tubes to establish electrical continuity. An external constant voltage power supply was connected to the Ag/AgCl electrodes for stimulation (Babona-Pilipos et al., 2011). Cells were electrically stimulated for 3 h with a dCEF strength of 250 mV/mm and electrical current between 1 and 1.5 mA (Babona-Pilipos et al., 2012). These parameters can promote cell migration (Babona-Pilipos et al., 2012), and if used in a therapy to promote cell migration, it is important to understand its effects on other cell behaviors such as proliferation.

Following stimulation, cells were collected, gently triturated, counted, and plated in 24-well plates in supplemented SFM. They were then cultured for 7 d, and the number of neurospheres >80 μm in diameter and the number of colonies 50–80 μm in diameter were counted (Reynolds and Weiss, 1992; Morshead et al., 1994; Piccin and Morshead, 2011).

For conditioned media (CM) experiments, cells from primary cultures or neurosphere-derived cells obtained from passaged neurospheres were plated and stimulated as above, and the CM was collected from stimulated and unstimulated conditions. Media were filtered through a 40-µm filter, and the resulting CM was diluted five times with supplemented SFM. The CM was added to primary or passaged cells plated at clonal density and cultured for 7 d. The number of neurospheres was then assessed.

### Differentiation

To assess cell differentiation, individual neurospheres were plated into 48-well plates coated with 25 μl of laminin dissolved in 5-ml SFM, for 4 h. Individual neurospheres >80 μm were placed into wells with 250 μl of 1% FBS in SFM (one sphere/well). After 7 d, cells were fixed with 4% paraformaldehyde (PFA) for 10 min following 3 × 5 min washes in 1× PBS. Cells were triple stained using a protocol adapted from Babona-Pilipos et al. (2011). Briefly, cells were washed 3 × 5 min with 1 × PBS, blocked for 1 h at room temperature with 10% normal goat serum in 1 × PBS, and incubated with O4 mouse monoclonal IgM (oligodendrocytes; 1:1000; R&D Systems MAB1326) in 10% goat serum in 1 × PBS overnight at 4°C. The following day, cells were washed 3 × 5 min with 1 × PBS and incubated with goat anti-mouse IgM 568 (1:500; Invitrogen A110440) for 1 h. To stain for neurons and astrocytes, cells were washed 3 × 5 min in 1 × PBS, permeabilized with 0.3% Triton X-100 for 20 min and then blocked with 10% goat serum in PBS for 1 h at room temperature, followed by incubation with βIII tubulin rabbit polyclonal IgG (neurons; 1:1000; Biolegend Poly18020) and GFAP mouse polyclonal IgG (astrocytes; 1:500; Sigma G3893). Cells were incubated overnight at 4°C. The following day, cells were washed 3 × 5 min with 1 × PBS and incubated with goat anti-rabbit IgG 647 (1:500; Invitrogen A21245) and goat anti-mouse IgG 488 (1:500; Invitrogen A11001) for 1 h, followed by an additional 3 × 5 min with 1 × PBS. DAPI (1:10,000; Invitrogen D1306) was used for nuclear staining for 5 min, followed by a final 3 × 5 min 1 × PBS wash. Images were taken on an Olympus FV1000 laser scanning microscope at 20× magnification. The number of DAPI-labeled cells were counted over three fields of view (with a minimum of 100 cells counted/neurosphere) and averaged over six technical replicates per condition from three independent experiments.

### Symmetric division assay

To block symmetric divisions of neural stem cells, 0.2 µg/ml of recombinant mouse secreted Frizzled Related Protein 2 (sFRP2; R&D Systems, 1169-FR-025) was added to neurosphere-derived cell cultures in the presence or absence of an applied EF (Piccin and Morshead, 2011). After stimulation, cells were collected, counted, and re-plated in supplemented SFM for 7 d. The number of neuropheres were counted. Controls included stimulated and unstimulated cells without sFRP2.

### Electrode construction and implantation

The 3D-printed electrode base was designed in SOLIDWORKS 2017, and electrodes were prepared as described (Morrison et al., 2019). The cortical stimulating electrodes were made with 127-µm diameter platinum wire and were 2 mm long and 2 mm apart. The striatal electrodes were made with uninsulated 127-µm diameter platinum wires with a 2-mm-long medial lead and a 3.6-mm-long lateral lead that were 1.8 mm apart. In all *in vivo* applications, mice received implants 2 d before stimulation. Mice were placed in a stereotactic apparatus and an incision was made along the midline of the scalp. Using a dental drill (P/N 8177 #77, 0.018”, Kopf), two holes were drilled at anterior/posterior +0.8 mm, and medial/lateral −0.7 and −2.7 mm, relative to bregma (Paxinos and Franklin, 2004). The electrodes were lowered into the brain with forceps and the device was secured in place using Insta-cure+ cyanoacrylate glue (Bob Smith Industries). The skin was sutured with 4–0 sterile Sofsilk sutures (2297-VS881, Medtronic). Following the procedure, mice were housed individually in clean cages and placed under a heating lamp to recover. The mice received ketoprofen (5.0 mg/kg, s.c.) on completion of the procedure and at 24 h postsurgery.

### *In vivo* electrical stimulation

Mice were anesthetized with 1.5–2.5% isoflurane, and the implanted electrode was interfaced with biphasic electrical stimulator (Popovic and Keller, 2005; Morshead et al., 2018) with pulse parameters: <200 μA, with a ∼500-mV cathodal pulse amplitude and a ∼125-mV anodal pulse amplitude, at 500- and 2000-μs pulse widths, respectively, followed by a 1000-μs resting phase (Morrison et al., 2019; Iwasa et al., 2019) for a total time of 1 h. Following stimulation, mice were returned to their cages and killed 1 or 3 d later. For tissue analysis, mice were transcardially perfused with ice-cold PBS followed by 4% PFA for tissue analysis, or cervically dislocated and processed for the neurosphere assay as described Neurosphere assay section.

### Proliferation and immunohistochemistry

To label proliferating cells, mice received a single injection of the thymidine analog ethynyl deoxyuridine (EdU; 50 mg/kg in PBS, i.p.) at the time of stimulation. After 24 h, mice were perfused with 4% PFA, and the brains were removed and postfixed for 4 h, then transferred to 30% sucrose before sectioning. Brains were cryosectioned (ThermoScientific HM525) at 20 µm and placed onto Superfrost Plus Microscope Slides (Fisher Scientific 12-550-15). EdU visualization was performed using the Click-It kit with 647 Azide (ThermoFisher C10419) per the manufacturer’s instructions. Antibody staining was performed before EdU labeling. Sections were permeabilized with 0.3% Triton X-100 for 20 min at room temperature, blocked with either 10% normal goat serum or 5% normal donkey serum in 1 × PBS for 1 h at room temperature, and then stained with a cocktail of primary antibodies against Sox2 mouse polyclonal IgG (1:1000; Abcam AB97959), Iba1 rabbit polyclonal IgG (1:500; Wako 019-19741), and doublecortin (DCX) mouse monoclonal IgG_1_ (1:400; Santa Cruz sc-271390) in blocking serum, and incubated overnight at 4°C. Secondary antibodies used were goat anti-mouse IgG 488 (Invitrogen, A11001), goat anti-rabbit IgG 568 (Invitrogen, A11036), and donkey anti mouse IgG 568 (Invitrogen, 10037), all at 1:500 in PBS. DAPI (Invitrogen, D1306) was used for nuclear staining (1:10,000 in 1 × PBS for 5 min). Slides were coverslipped with Mowiol mounting media.

### Image analysis

A total of 15–28 sections from three to seven mice per condition were analyzed for each investigation. Imaging was performed with an Olympus FV1000 laser scanning microscope at 20× or 40× magnification to generate 20-μm-thick z-stacks. Images were taken of the dorsolateral corner of the lateral ventricle subependyma between the crossing of the anterior commissure and the genu of the corpus callosum. A 600-μm^2^ (DAPI&EdU) or 350-μm^2^ (EdU&Sox2, EdU&DCX, and EdU&Iba1) region of interest was examined in each section ipsilateral to the electrode implantation using Fiji Imaging Software (Schindelin et al., 2012). A smaller region was used in the latter condition to maximize the proportion of positive cells viewed in each condition. The total number of nuclei (DAPI+) were counted and the number of labeled cells (Sox2, DCX, Iba1, and EdU) were quantified and expressed as a percent of total DAPI+ cells.

### Fluorescent activated cell sorting

GFAP::GFP mice were implanted with electrodes and stimulated as described. A total of five mice were used for each group, per experiment. FACS analysis was performed as previously described (Codega et al., 2014) to isolate activated versus quiescent neural stem cell populations. Briefly, the periventricular region was dissected, digested with papain (10 min at 37°C; Worthington) in PIPES solution [120 mm NaCl, 5 mm KCl, 50 mm PIPES (Sigma), 0.6% glucose, 1× Pen/Strep (Invitrogen) in H_2_O, pH adjusted to 7.6] and mechanically dissociated to single cells after adding ovomucoid (Worthington, 0.7 mg/5 mice) and DNase (Worthington, 1000 U/5 mice) in 5-ml total volume. Cells were centrifuged for 10 min at 4°C in 22% Percoll (Sigma) to remove myelin and incubated for 15 min with A647-complexed EGF (1:300; Invitrogen) and biotinylated rat anti-mCD133 (1:300, clone 13A4, eBioscience), washed by centrifugation, and incubated for 15 min with PE-Cy7-conjugated streptavidin (1:1000; eBioscience). All staining and washes were conducted on ice in a solution of 1% BSA, 0.1% glucose HBSS. Cell viability was assessed with DAPI (1:10,000) in 1 × PBS before sorting. Cells were sorted using a Becton Dickinson Influx or FACS Aria II apparatus using 13 PSI pressure and 100-µm nozzle aperture. The gating strategy is shown in Extended Data Figure 3-1. Data were analyzed with FlowJo 9.3 software (BD Life Sciences) and displayed using biexponential scaling (Codega et al., 2014).

**Figure 1.**
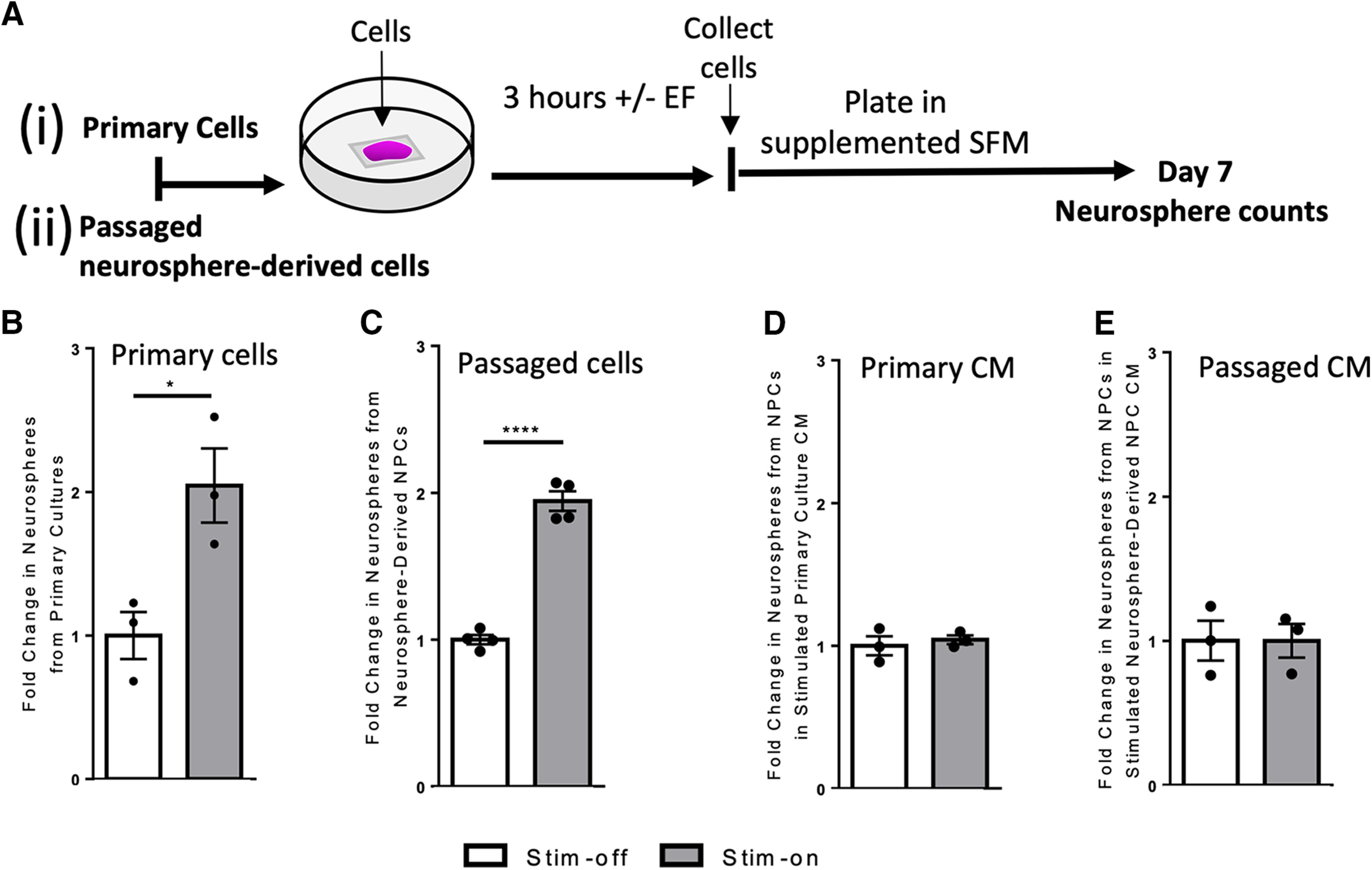
*In vitro* electrical stimulation increases the number of neurospheres. ***Ai***, ***Aii***, Experimental paradigms for *in vitro* electrical stimulation. ***B***, ***C***, Fold change in the number of neurospheres from stim-off cells and stim-on cells from (***B***) primary cultures (*n* = 3 independent experiments, *p = 0.03) and (***C***) neurosphere-derived NPCs (*n* = 4 independent experiments, ****p < 0.0001). Fold change in the number of neurospheres cultured using CM from stim-off and stim-on (***D***) primary cells (*n* = 3 independent experiments) and (***E***) neurosphere-derived NPCs (*p* = 0.5 and *p* = 0.9, respectively; *n* = 3 independent experiments) Each point in the graph represents an independent experiment, plotted with mean ± SEM. Analysis performed via a two-tailed unpaired *t* test between stim-off and stim-on groups. Neurosphere counts from *in vitro* stimulation found in Extended Data Figure 1-1. Comparison of fold changes in neurospheres following stimulation with and without Wnt inhibitor to inhibit symmetric divisions found in Extended Data Figure 1-2.

10.1523/ENEURO.0273-20.2020.f1-1 Extended Data Figure 1-1 Neurosphere counts from *in vitro* stimulation. Data reported in mean ± SEM; *n* = 3–4 mice per group. Download Figure 1-1, DOC file.

10.1523/ENEURO.0273-20.2020.f3-1 Extended Data Figure 3-1 Gating strategy for separating GFAP::GFP^+^CD133^+^EGFR^high^ cells from GFAP::GFP^+^CD133^+^EGFR^low^ cells adapted from Codega et al. (2014). ***A***, Cell debris was removed and NPCs were sorted using forward scatter (FSC-H) for cell size and side scatter (SSC-H) for cell granularity. ***B***, Single, live cells were gated by removing doublets (***C***) and (***D***) DAPI^–^ cells. ***E***, GFP+ cells from the GFAP::GFP^+^ were selected and three populations were defined: GFAP::GFP^+^CD133^–^ (red), GFAP::GFP^+^CD133^+^EGFR^low^ (blue) and GFAP::GFP^+^CD133^+^EGFR^high^ (green). Age matched wild-type C57BL/6J mice were used for single color-stained cells and fluorescent minus one (FMO) gating optimization in which GFP was not required. Download Figure 3-1, TIF file.

### Statistical analysis

All data are reported as mean ± SEM unless otherwise indicated. Statistical analysis was performed using GraphPad Prism 6 (GraphPad). For comparisons between multiple groups, ANOVA followed by Tukey’s *post hoc* test was used, as indicated. For comparisons between two groups, two-tailed unpaired Student’s *t* tests were used; *p* < 0.05 was regarded as statistically significant.

## Results

### *In vitro* electrical stimulation increases the number of neural stem cells

To examine the effects of electrical stimulation on NPCs, we first performed *in vitro* experiments using the neurosphere assay. Neurospheres are free-floating colonies consisting of a minority of stem cells and a majority of progenitors (Reynolds and Weiss, 1992; Morshead et al., 1994). In clonal conditions, the number of spheres over 80 µm gives the number of neural stem cells in the brain (Coles-Takabe et al., 2008). Tissue dissected from the adult mouse periventricular region was plated in a stimulation chamber to evaluate the effect of an applied EF on cell behavior. The primary dissected brain tissue (containing NPCs and the niche cells) were exposed to no electrical stimulation (stim-off, control) or electrical stimulation (stim-on) of ∼250 mV/mm for 3 h (Fig. 1*Ai*). Cells were then collected from the chamber and re-plated at clonal density, cultured for 7 d, and the number of neurospheres >80 μm (derived from multipotent, self-renewing stem cells; Coles-Takabe et al., 2008) were counted (Fig. 1*Ai*). We observed a 2.0-fold increase in the total number of neurospheres following electrical stimulation compared with unstimulated cells (*p* = 0.03; Fig. 1*B*; Extended Data Fig. 1-1). We observed a similar 2.0-fold increase in the total number of neurospheres between stim-off and stim-on groups following EF application to passaged neurosphere-derived cells (i.e., in the absence of the niche cells; *p* < 0.0001; Fig. 1*C*; Extended Data Fig. 1-1). Hence, the size of the neural stem cell pool is expanded in response to electrical stimulation.

### Secreted factors are not sufficient for electrical stimulation induced neural stem cell expansion

We next asked whether factors released from EF-exposed cells were mediating the increase in the neural stem cell pool after stimulation. We took passaged NPCs and plated them in CM collected from stimulated primary cultures. There was no change in the number of primary neurospheres in the presence of CM, regardless of stimulation (*p* = 0.5; Fig. 1*D*; Extended Data Fig. 1-1), suggesting that released factors from niche cells were not mediating the expansion. The same experiment was performed using CM from passaged NPCs under stim-off and stim-on conditions, and again we observed no effect on the size of the neural stem cell pool (*p* = 0.9; Fig. 1*E*; Extended Data Fig. 1-1). Taken together, these results reveal that secreted factors from primary niche cells or NPCs themselves following electrical stimulation are not driving the expansion in the size of the neural stem cell pool.

### Electrical stimulation leads to increased cell survival and differences in differentiation

One mechanism that can lead to expansion of the stem cell pool is an increase in symmetric cell division, wherein a single stem cell divides to give rise to two stem cells (as opposed to asymmetric division that produces a single stem cell and a progenitor cell). Wnt signaling has been shown to promote symmetric division of adult neural stem cells (Piccin and Morshead, 2011); hence, we inhibited Wnt signaling during electrical stimulation to block symmetric division and assessed the effect on neurosphere numbers. Using the *in vitro* paradigm shown in Figure 1*Aii*, cells were placed in stim-on or stim-off conditions for 3 h in the presence or absence of the Wnt3a antagonist, sFRP2, then collected and cultured in neurosphere conditions. We observed a similar increase in neurosphere formation following stimulation, with or without inhibitor (*p* = 0.3; Extended Data Fig. 1-2). Hence, enhanced symmetric stem cell division through the Wnt signaling pathway is not sufficient to account for the stimulation induced expansion in the size of the neural stem cell pool.

**Figure 2:**
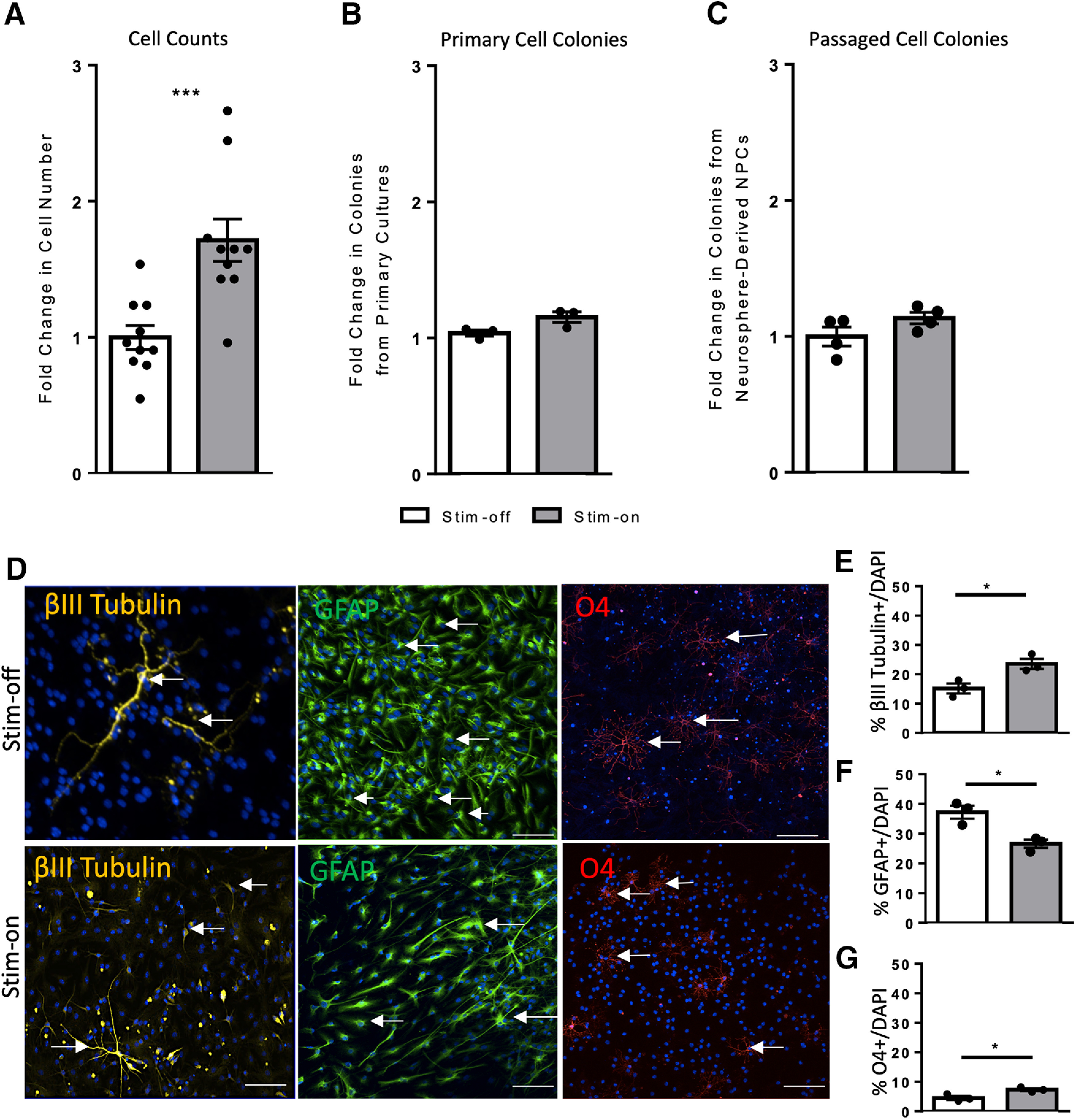
*In vitro* electrical stimulation increases cell number and regulates NPC differentiation. ***A***, A 1.7-fold increase in the number of living cells is seen following stim-on when compared with the stim-off cells processed the same way (62,400 ± 5705 vs 36,400 ± 3205 cells, stim-on vs stim-off respectively; *n* = 10 individual experiments; ***p = 0.0009). ***B***, ***C***, Fold change in the number of colonies from stim-off and stim-on in (***B***) primary cells (*p* = 0.06; *n* = 3 independent experiments) and (***C***) neurosphere-derived NPCs (*p* = 0.1; *n* = 4 independent experiments). Neurosphere counts from *in vitro* stimulation found in Extended Data Figure 1-1. ***D***, Differentiated neurospheres stained for βIII tubulin (neurons), GFAP (astrocytes), and O4 (oligodendrocytes). White arrows indicate examples of cells co-localized with DAPI (blue). Scale bar = 100 µm. ***E–G***, Quantification of cells as a percent of DAPI (*n* = 10 spheres/treatment, 3 mice per group) expressing (***E***) βIII tubulin (*p = 0.02), (***F***) GFAP (*p = 0.01), and (***G***) O4 (*p = 0.03). Each point in the graph represents an independent experiment, plotted with mean ± SEM. All analysis performed via a two-tailed unpaired *t* test between stim-off and stim-on.

We hypothesized that electrical stimulation was promoting NPC survival, which could account for the increased numbers of neurospheres in stim-on conditions. To test this, we assessed the total number of live cells via Trypan Blue exclusion and demonstrated a 1.7-fold increase in the number of live cells following stimulation, when compared with the non-stimulated cells processed the same way (62,400 ± 5705 vs 36,400 ± 3205 cells, stim-on vs stim-off, respectively; *p* = 0.0009; Fig. 2*A*). This supports the hypothesis that electrical stimulation promotes cell survival.

Since neurospheres >80 μm in diameter are derived from stem cells, the applied EF was enhancing stem cell survival. To determine whether electrical stimulation resulted in increased neurosphere formation from progenitor cells, the number of 50–80 μm in diameter colonies (progenitor-derived) were counted. The number of these colonies was similar (Fig. 2*B*,*C*; Extended Data Fig. 1-1) under stim-on conditions compared with stim-off in primary cells (*p* = 0.06) and passaged neurosphere-derived cells (*p* = 0.1).

We next sought to determine whether electrical stimulation alters the differentiation profile of neurosphere-derived cells. Neurospheres from stim-off and stim-on cultures were collected and plated in differentiation conditions for 7 d, and immunohistochemistry was performed to assess the number of neurons (βIII tubulin+), astrocytes (GFAP+), and oligodendrocytes (O4+) as a percent of DAPI+ nuclei (Fig. 2*D*). Neurospheres from stim-on conditons gave rise to 1.5-fold increase in neurons compared with stim-off controls (23.3 ± 1.7% vs 15.1 ± 1.6% βIII tubulin+, stim-on vs stim-off, respectively; *p* = 0.02; Fig. 2*E*) and a 0.7-fold decrease in astrocyte formation (26.6 ± 1.4% vs 37.2 ± 2.2% GFAP+, stim-on vs stim-off, respectively; *p* = 0.01; Fig. 2*F*). Finally, a 1.6-fold increase in oligodendrocytes was observed following electrical stimulation (7.3 ± 0.5% vs 4.5 ± 0.7% O4+, stim-on vs stim-off, respectively; *p* = 0.03; Fig. 2*G*). Hence, electrical stimulation modified the differentiation profile of NPCs by promoting neurogenesis and oligogenesis and decreasing astrocyte formation.

### *In vivo* cortical stimulation expands the size of the neural stem cell pool

**Figure 3.**
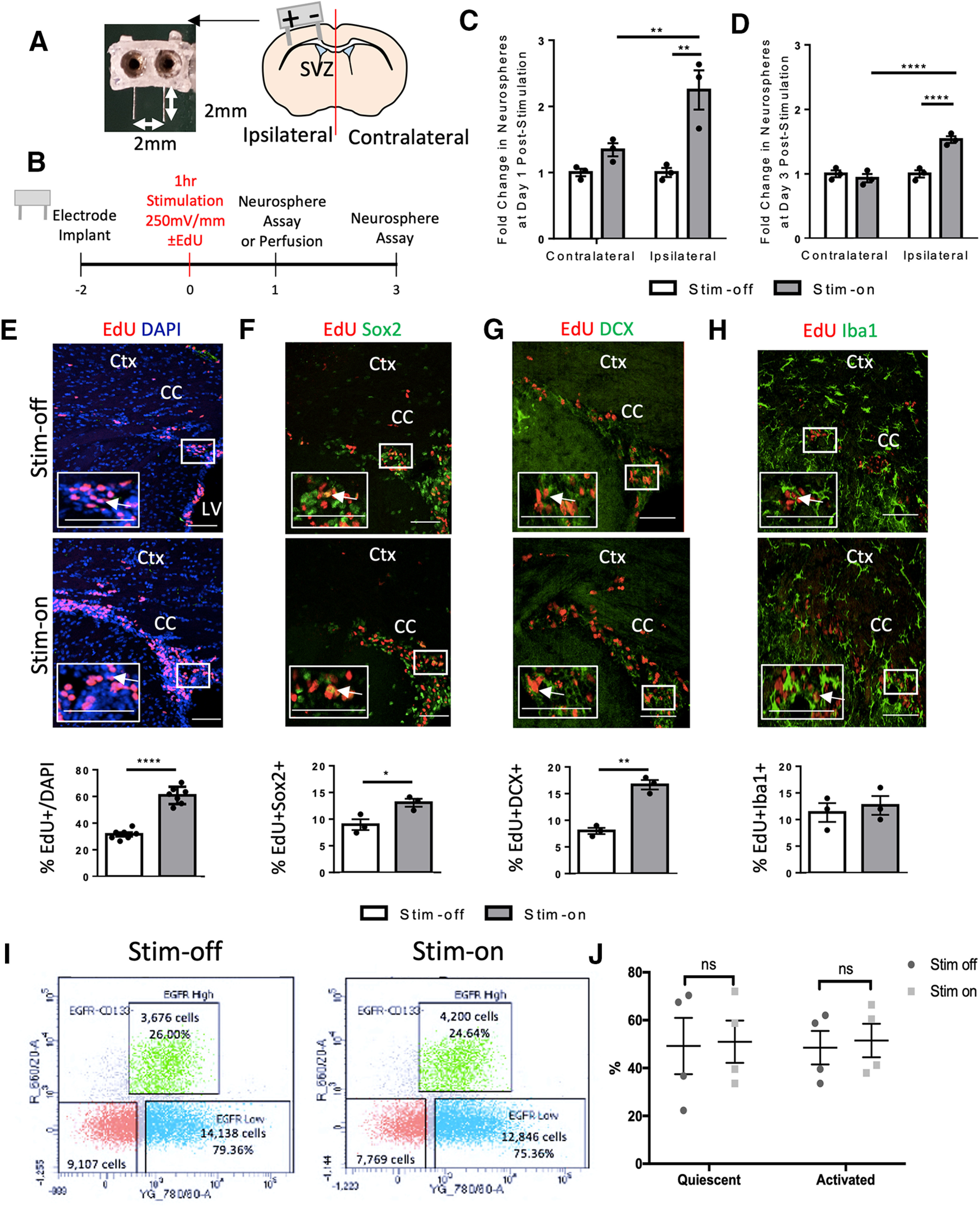
*In vivo* electrical stimulation increases the number of neurospheres and proliferation by a means other than activation. ***A***, Image of 3D-printed electrode and schematic of a coronal section through the forebrain indicating the electrode implantation. Anode (+) and cathode (–) are shown. SVZ, subventricular zone. ***B***, Experimental paradigm. ***C***, ***D***, Fold change in the number of neurospheres from stim-off and stim-on brains in the ipsilateral and contralateral hemisphere at (***C***) day 1 poststimulation (**p < 0.04) and (***D***) day 3 poststimulation [****p < 0.0001; *n* = 3 mice/group, two-way ANOVA (α = 0.05) with Tukey’s *post hoc* analysis]. There was no difference in fold change between days 1 and 3 as seen in Extended Data Figure 3-3. Neurosphere counts from *in vivo* stimulation found in Extended Data Figure 3-2. ***E–H***, Images of the dorsolateral corner of the lateral ventricle ipsilateral to the electrode in 600 µm^2^ (EdU&DAPI) or 350 µm^2^ (EdU&Sox2, EdU&DCX, EdU&Iba1) and quantification. White arrows indicate cells positive for both labels in each image. Ctx, cortex; LV, lateral ventricle; CC, corpus callosum. White box indicates high power inset of dorsolateral corner (or corpus callosum for Iba1&Edu) in bottom left of image. Scale bar =100 µm (EdU&DAPI) or 50 µm. Quantification of cells as a percent of DAPI in the region of interest for stim-off and stim-on brains for (***E***) EdU+ cells (****p < 0.0001; *n* = 7 mice/group), (***F***) EdU+Sox2+ cells (*p = 0.03; *n* = 3 mice/group), (***G***) EdU+DCX+ cells (**p = 0.001; *n* = 3 mice/group), and (***H***) EdU+Iba1+ cells (*p* = 0.6; *n* = 3 mice/group). Analysis performed via two-tailed *t* test unpaired *t* test between stim-off and stim-on groups. ***I***, Contour plots showing cell sorts from the stim-off and stim-on brains with the gating strategy as seen in Extended Data Figure 3-1. ***J***, The relative percentage of activated and quiescent cells (*n* = 4 independent experiments, 5 mice per experiment). The green boxes in the contour plots represent GFAP::GFP^+^CD133^+^EGFR^high^ cells, blue represents GFAP::GFP^+^CD133^+^EGFR^low^ cells, and red represents GFAP::GFP^+^CD133^–^ cells (two-way ANOVA, α = 0.05 with Tukey’s *post hoc* multiple comparison test). Each point in the graphs represents an independent experiment, plotted with mean ± SEM. ns indicates there was no significance in quiescent to activated shift in either stim off or stim on group.

In the next series of experiments, we asked whether *in vivo* electrical stimulation had similar effects on endogenous NPCs in the periventricular region of the adult forebrain. Stimulating electrodes were implanted into the cortex with the cathode placed near the midline and the anode placed laterally, as previously reported (Morrison et al., 2019; Iwasa et al., 2019; Fig. 3*A*). Two days postimplantation, mice received electrical stimulation for 1 h (250 mV/mm during the cathodal pulse of the asymmetric biphasic stimulation; Fig. 3*B*), a similar duration to previous reports examining proliferation in the hippocampus (Stone et al., 2011). At 1 d poststimulation, the neurosphere assay was performed from the periventricular regions of each hemisphere. Consistent with our *in vitro* findings, we observed a 2.3-fold increase in the number of neurospheres from the ipsilateral hemisphere, compared with the contralateral hemisphere, in stim-on brains (*p* = 0.04; Fig. 3*C*; Extended Data Fig. 3-2). To rule out an effect of the implantation procedure itself (Sachewsky et al., 2014; Obernier et al., 2018), we compared with stim-off brains and found no difference in neurosphere formation (*p* > 0.9999; Fig. 3*C*; Extended Data Fig. 3-2) between the contralateral and ipsilateral hemispheres.

10.1523/ENEURO.0273-20.2020.f3-2 Extended Data Figure 3-2 Neurosphere counts from *in vivo* cortical stimulation. Data reported in mean ± SEM; *n* = 3 mice per group. Download Figure 3-2, DOC file.

10.1523/ENEURO.0273-20.2020.f3-3 Extended Data Figure 3-3 Changes in the ipsilesional hemispheres from stim off and stim on. There was no difference in fold change between days 1 and 3; *n* = 3 mice per group, two-tailed unpaired *t* test, *p* = 0.1. Download Figure 3-3, TIF file.

We found that the expansion of the neural stem cell pool persisted for at least 3 d poststimulation as we observed a 1.5-fold increase in neurosphere formation in the ipsilateral versus contralateral hemispheres of stim-on brains (*p* = 0.002; Fig. 3*D*; Extended Data Fig. 3-2). Again, there was no significant difference between the contralateral hemispheres of stim-on versus stim-off brains (*p* = 0.5; Fig. 3*D*; Extended Data Fig. 3-2) or the ipsilateral stim-off hemisphere (*p* = 0.5; Fig. 3*D*; Extended Data Fig. 3-2), revealing that electrode implantation alone did not account for the expansion. The magnitude of the neural stem cell pool expansion was not different between 1 and 3 d poststimulation (*p* = 0.08; Extended Data Fig. 3-3). Hence, electrical stimulation alters the kinetics of neural stem cells *in vivo*, leading to an expansion in the size of the neural stem cell pool.

We next examined the brain section using immunohistochemistry to assess *in vivo* whether there was a concomitant expansion in NPCs and differentiated progeny. We used Sox2 to label NPCs along with the proliferation marker EdU. Mice received an injection of EdU at the time of stimulation and were killed 1 d later (Fig. 3*B*). The number of EdU+ cells were counted in the dorsolateral corner of the lateral ventricle and revealed a significant increase in the relative number of proliferating cells as a result of stimulation (69.0 ± 1.9% vs 31.5 ± 1.5% EdU+ cells, stim-on vs stim-off, respectively; *p* < 0.0001; Fig. 3*E*). We observed a concomitant increase in the number of Sox2+ cells (27.4 ± 1.1% vs 22.1 + 0.7% cells, stim-on vs stim-off, respectively; *p* = 0.02) and an increase in the percentage of EdU+Sox2+ cells (13.1 ± 0.7% vs 9.0 ± 1.05% Sox2+EdU+ cells, stim-on versus stim-off, respectively; *p* = 0.03) in the ipsilateral hemisphere following stimulation (Fig. 3*F*). To further assess the phenotype of EdU+ cells, we used the neuroblast marker DCX and microglia marker Iba1 (Fig. 3*G*,*H*). Similar to our *in vitro* findings, we observed a significant increase in the proportion of both DCX+ (25.2 ± 3.9% vs 16.6 ± 2.7% cells, stim-on vs stim-of, respectively; *p* = 0.03) and EdU+DCX+ (16.7 ± 0.9 vs 8.0 ± 0.6 cells, stim-on vs stim-off, respectively; *p* = 0.001; Fig. 3*G*) cells following stimulation. The proportion of Iba1+ cells (25.9 ± 2.6% vs 29.4 ± 1.9% Iba1+ cells, stim-on and stim-off, respectively) was unaffected (*p* = 0.3), as was the proportion of proliferating microglia (Iba1+ EdU+; 12.7 ± 1.8% vs 11.3 ± 1.8% Iba1+EdU+ cells, stim-on and stim-off, respectively; *p* = 0.6; Fig. 3*H*) similar to previous findings (Morrison et al., 2019; Iwasa et al., 2019). Taken together, our findings reveal that electrical stimulation alters the proliferation and differentiation of NPCs but not the microglia numbers *in vivo*.

### Cortical EF application does not activate quiescent stem cells

Within the brain, neural stem cells exist in two states: quiescent and activated (Codega et al., 2014; Llorens-Bobadilla et al., 2015; Morizur et al., 2018; Kobayashi et al., 2019). Quiescent neural stem cells do not produce neurospheres within 7 d *in vitro*; therefore, an increased neurosphere formation following EF application could reflect the conversion of a quiescent stem cell to an activated, neurosphere forming state. If true, we predicted that electrical stimulation would change the ratio of activated:quiescent stem cells from stimulated brains. Hence, we performed FACS on cells derived from the adult periventricular zone of stim-off and stim-on treated brains. Neural stem cells express GFAP (Morshead et al., 2003; Liu et al., 2010; Codega et al., 2014) hence GFAP::GFP mice were used for the analyses. The surface marker CD133 was used to further delineate neural stem cells, while the epidermal growth factor receptor (EGFR) was used to represent activated stem cells (Codega et al., 2014). At 1 h following stimulation (or not) mice were killed, and cells were sorted based on GFP expression, CD133^+^ (precursor cell marker) and EGF receptor (EGFR^+^) for activated versus quiescent cells (GFAP^+^CD133^+^EGFR^+^ vs GFAP^+^CD133^+^EGFR^−^; activated vs quiescent; Codega et al., 2014). The green in the contour plots represent EGFR^high^ CD133^+^ cells, blue represents EGFR^low^ CD133^+^ cells, and red represents EGFR^−^CD133^−^ cells. We found no significant difference in the relative ratio of activated:quiescent stem cells in the stim-off and stim-on groups (Fig. 3*I*,*J*), suggesting that electrical stimulation does not lead to the activation of quiescent stem cells *in vivo.*

### Modified EF application results in expansion of the neural stem cell pool

**Figure 4.**
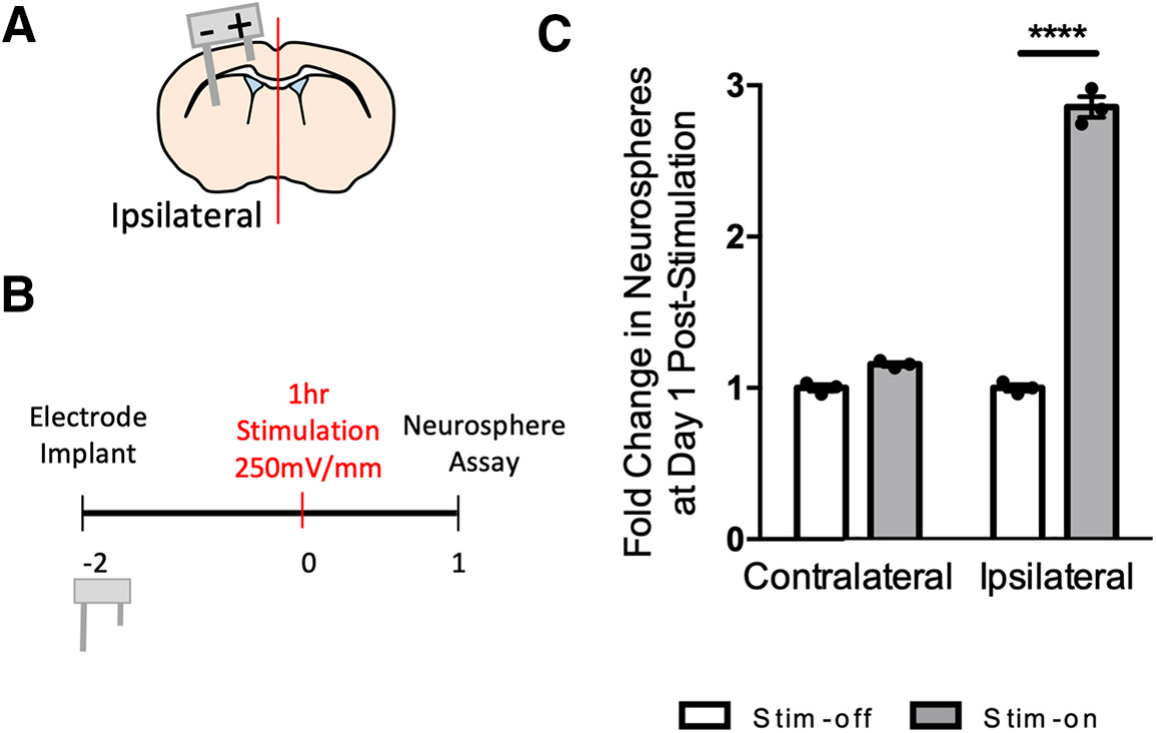
Striatal electrodes increase the number of neurospheres. ***A***, Schematic of striatal electrodes in brain. Anode (+) and cathode (−). ***B***, Experimental paradigm. ***C***, Fold change in the number of neurospheres from stim-off and stim-on brains in the ipsilateral hemisphere following striatal stimulation (****p < 0.0001; *n* = 3 mice/group, two-tailed *t* test between each stim-off and stim-on groups). Each point in the graphs represents an independent experiment, plotted with mean ± SEM. Neurosphere counts from *in vivo* striatal stimulation are found in Extended Data Figure 4-1.

We asked whether changing the configurations of the electrodes impacts the efficacy of the neural stem cell expansion by altering the EF distribution (Butson and McIntyre, 2005). The length of the lateral electrode was increased to reach the striatum resulting in the EF encompassing the periventricular niche as it is directly between the two leads (Fig. 4*A*). The number of neurospheres was assessed 1 d poststimulation in the ipsilateral hemispheres of stim-off versus stim-on brains (Fig. 4*B*). The striatal electrode design resulted in a 2.9-fold increase in the size of the neural stem cell pool in stim-on brains (*p* < 0.0001; Fig. 4*C*; Extended Data Fig. 4-1). There was no difference in the contralateral hemispheres of stim-off and stim-on brains (*p* = 0.1; Fig. 4*C*; Extended Data Fig. 4-1). These findings demonstrate that striatal DBS results in neural stem cell expansion and highlights the potential to optimize the parameters to regulate NPC behavior.

10.1523/ENEURO.0273-20.2020.f4-1 Extended Data Figure 4-1 Neurosphere counts from *in vivo* striatal stimulation. Data reported in mean ± SEM; *n* = 3 mice per group. Download Figure 4-1, DOC file.

## Discussion

We examined the proliferation kinetics and differentiation profile of NPCs following short EF application using both *in vitro* and *in vivo* approaches. This work builds on our previous studies demonstrating that NPCs are electrosensitive cells that undergo rapid and directed migration in response to electrical stimulation (Babona-Pilipos et al., 2011, 2015; Iwasa et al., 2019). We show that electrical stimulation can expand the size of the neural stem cell pool and increase neurogenesis, both *in vitro* and *in vivo.* Moreover, we have demonstrated that NPCs in the neurogenic periventricular region of the adult brain can be regulated by changing the electrode design which modifies the EF application to the neural stem cell niche.

Our *in vitro* findings reveal that the stimulation-dependent increase in neurosphere numbers is because of its actions on neural stem cells, and not mediated by factors released by the niche or NPCs in response to EF application. We provide evidence that the expansion is because of increased survival rather than through increased symmetric division, which has previously been shown to enhance the size of the adult neural stem cell pool (Piccin and Morshead, 2011; Piccin et al., 2014). Indeed, while the Wnt signaling pathway is important in promoting symmetric division of adult neural stem cells (Adachi et al., 2007; Piccin et al., 2011, 2014; Azim et al., 2014; Edri et al., 2015), we demonstrate that blocking the Wnt signaling pathway was not sufficient to abolish the increase in neurospheres seen with electrical stimulation compared with control. Our findings were consistent with electrical stimulation promoting stem cell survival (Du et al., 2018), with the potential to provide an effective approach to enhance neural regeneration. Interestingly, DBS has been used in clinical setting for many years to treat advanced stages of PD, despite a paucity of knowledge related to the functional substrates that lead to benefits. Neuronal loss is a prominent feature of PD and the possibility that DBS can enhance neural stem cell survival, promote neurogenesis and NPC migration (Cao et al., 2013; Feng et al., 2017; Iwasa et al., 2019) may provide some context for the success of DBS.

We found the effects of electrical stimulation were not limited to changes in neurosphere number. We demonstrated increased neurogenesis and oligogenesis following electrical stimulation *in vitro*, similar to other reports (Ariza et al., 2010; Chang et al., 2016; Dong et al., 2019). Chang et al. (2016) demonstrated that embryonic murine NPCs produced increased oligodendrocytes (via O4+ cells) and neurons (via neuronal marker βIII+ cells) following direct current pulses of 300 mV/mm at 100 Hz *in vitro* after 7 d in culture. In adult rat NPCs derived from the dentate gyrus, Ariza et al. (2010) showed that application of a direct current EF of 437 mV/mm led to increased neuron production (βIII+ cells) compared with controls or alternating current EFs of 46 mV/mm. Dong et al. (2019) found increased neuronal differentiation *in vitro* following electrical stimulation as we did, and showed that knocking out the gene Ascl1, implicated in neuronal differentiation, abolished increases in neurogenic differentiation seen following electrical stimulation. These studies demonstrate the ability of electrical stimulation at a range of field strengths to regulate both proliferation and differentiation with the same electrical stimulation paradigm and highlight the need to optimize stimulation parameters depending on the desired outcome.

*In vivo*, our findings are consistent with reports of increased number of proliferating cells after electrical stimulation in the dentate gyrus, cortex, and third ventricle (Stone et al., 2011; Rueger et al., 2012; Vedam-Mai et al., 2014). We asked whether this expansion was a result of quiescent neural stem cells being activated and contrary to our prediction, we found that the ratio of activated to quiescent neural stem cells remained the same following stimulation. One possibility is we missed the change in activated neural stem cells, which could have occurred sooner than 24 h after stimulation. Interestingly, we observed that at 3 d poststimulation, there is still a significant increase in the size of the neural stem cell pool, suggesting the effects can be long term. With the overall goal of developing electrical stimulation as a therapeutic to promote endogenous NPC-mediated neural repair, investigating the early and late effects of EF application will be an important next steps toward understanding the cell-based mechanisms underlying the outcomes.

## Conclusion

We have shown that electrical stimulation *in vitro* and *in vivo* promotes a change in NPC behavior; expanding the size of the neural stem cell pool and enhancing neurogenesis. Our findings suggest that increased cell survival can account for these changes. This study provides insight into the effects of electrical stimulation on neural stem and progenitor cells and its potential to enhance neural repair.

10.1523/ENEURO.0273-20.2020.f1-2 Extended Data Figure 1-2 Comparison of fold changes in neurospheres following stimulation with and without Wnt inhibitor (sFRP2). ***A***, sFRP2 blocked the vast majority of neurosphere growth in the stim-off cultures as predicted with a 16-fold decrease of stem cells and (***B***) a 17-fold decrease in stim-on conditions (****p < 0.0001). ***C***, Blocking symmetric division does not inhibit the increase in neurospheres following electrical stimulation; *n* = 3 independent experiments, two-tailed unpaired *t* test, *p* = 0.3. Download Figure 1-2, TIF file.

## Synthesis

Reviewing Editor: Julie Andersen, Buck Institute Decisions are customarily a result of the Reviewing Editor and the peer reviewers coming together and discussing their recommendations until a consensus is reached. When revisions are invited, a fact-based synthesis statement explaining their decision and outlining what is needed to prepare a revision will be listed below. Note: If this manuscript was transferred from JNeurosci and a decision was made to accept the manuscript without peer review, a brief statement to this effect will instead be what is listed below.

Provides a solid description of changes in neural stem/progenitor cell pool in the adult SVZ following electrical field stimulation. The current study adds new information about this interesting phenomenon, which has been studied for some time.
