# Supplementary material for: Electric Field Application In Vivo Regulates Neural Precursor Cell Behavior in the Adult Mammalian Forebrain
Source: eNeuro. 2020 Aug 21;7(4):ENEURO.0273-20.2020. doi: 10.1523/ENEURO.0273-20.2020 (PMC7452733; doi:10.1523/ENEURO.0273-20.2020)
Supplement: Extended Data Figure 1-1 — Neurosphere counts from in vitro stimulation. Data reported in mean ± SEM; n = 3–4 mice per group. Download Figure 1-1, DOC file. [file enu-eN-NWR-0273-20-s02.doc]

**Figure 1-1: Neurosphere counts from *in vitro* stimulation**

| **Condition** | **Stim-off**  **(spheres/5,000 cells)** | **Stim-on**  **(spheres/5,000 cells)** |
| --- | --- | --- |
| Primary Culture | 1.5±0.2 | 3.0±0.4 |
| Neurosphere-Derived NPCs | 10.8±0.4 | 20.9±0.8 |
| NPCs in Stimulated Primary CM | 4.7±0.3 | 4.9±0.1 |
| NPCs in Stimulated Passaged CM | 4.5±0.6 | 4.2±0.3 |
| Colonies from Primary Culture | 7.1±0.2 | 8.5±0.5 |
| Colonies from Neurosphere-Derived NPCs | 15.0±1.0 | 17.0±0.6 |

Data are reported as mean ± SEM.
