# Supplementary figures and images for: Electric Field Application In Vivo Regulates Neural Precursor Cell Behavior in the Adult Mammalian Forebrain
Source: eNeuro. 2020 Aug 21;7(4):ENEURO.0273-20.2020. doi: 10.1523/ENEURO.0273-20.2020 (PMC7452733; doi:10.1523/ENEURO.0273-20.2020)

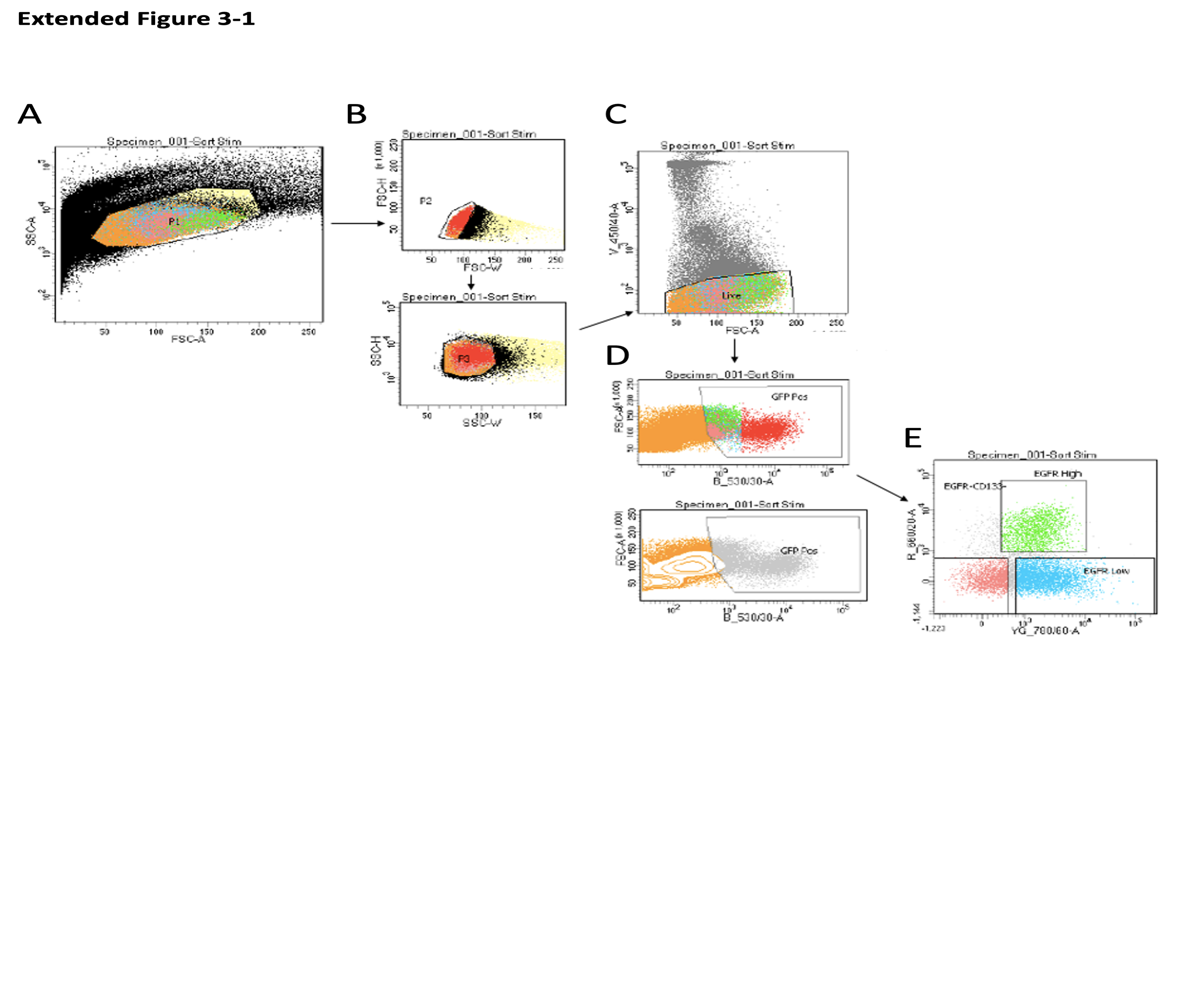

Supplement: Extended Data Figure 3-1 — Gating strategy for separating GFAP::GFP+CD133+EGFRhigh cells from GFAP::GFP+CD133+EGFRlow cells adapted from Codega et al. (2014). A, Cell debris was removed and NPCs were sorted using forward scatter (FSC-H) for cell size and side scatter (SSC-H) for cell granularity. B, Single, live cells were gated by removing doublets (C) and (D) DAPI– cells. E, GFP+ cells from the GFAP::GFP+ were selected and three populations were defined: GFAP::GFP+CD133– (red), GFAP::GFP+CD133+EGFRlow (blue) and GFAP::GFP+CD133+EGFRhigh (green). Age matched wild-type C57BL/6J mice were used for single color-stained cells and fluorescent minus one (FMO) gating optimization in which GFP was not required. Download Figure 3-1, TIF file. [file enu-eN-NWR-0273-20-s06.tif]

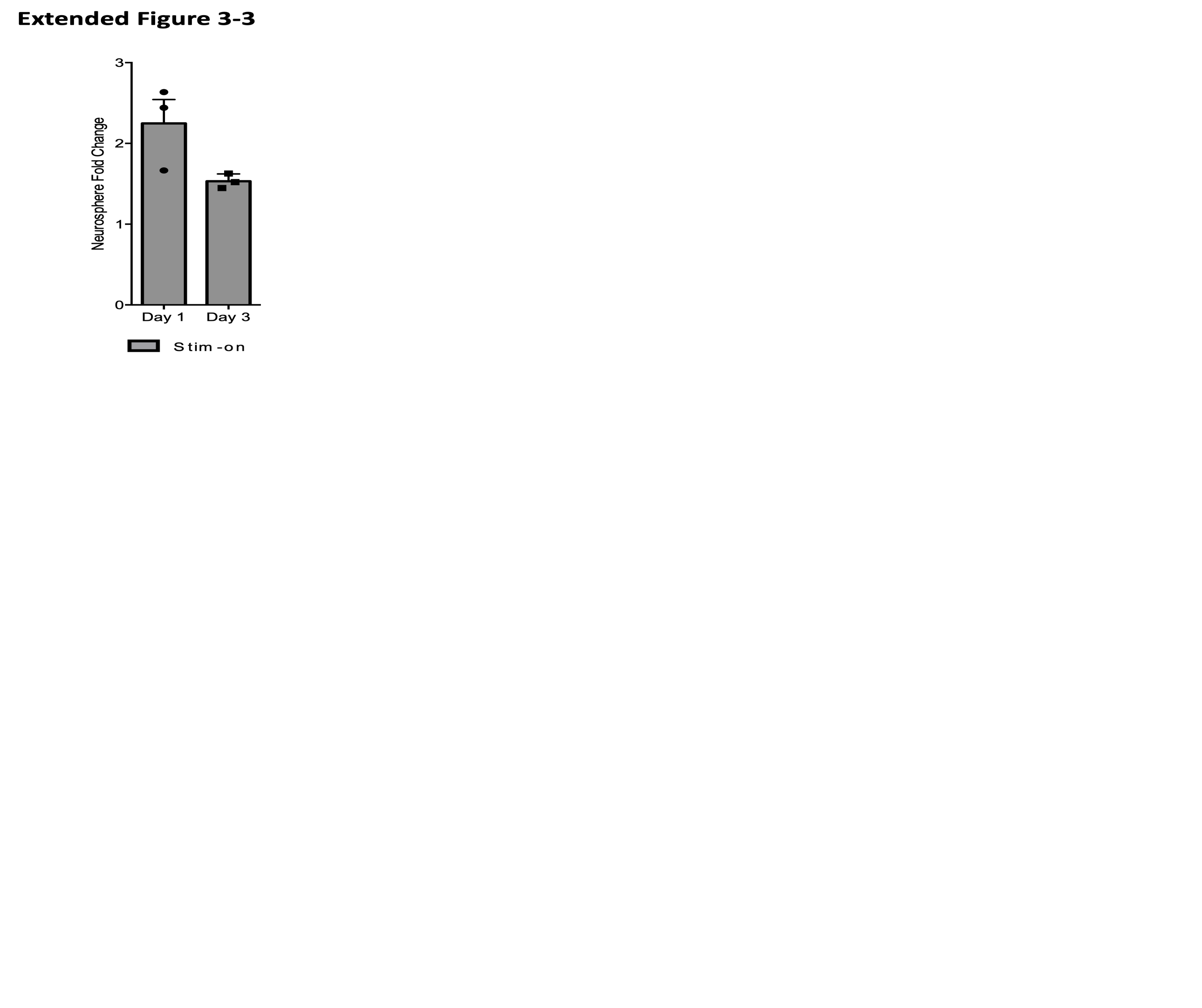

Supplement: Extended Data Figure 3-3 — Changes in the ipsilesional hemispheres from stim off and stim on. There was no difference in fold change between days 1 and 3; n = 3 mice per group, two-tailed unpaired t test, p = 0.1. Download Figure 3-3, TIF file. [file enu-eN-NWR-0273-20-s07.tif]

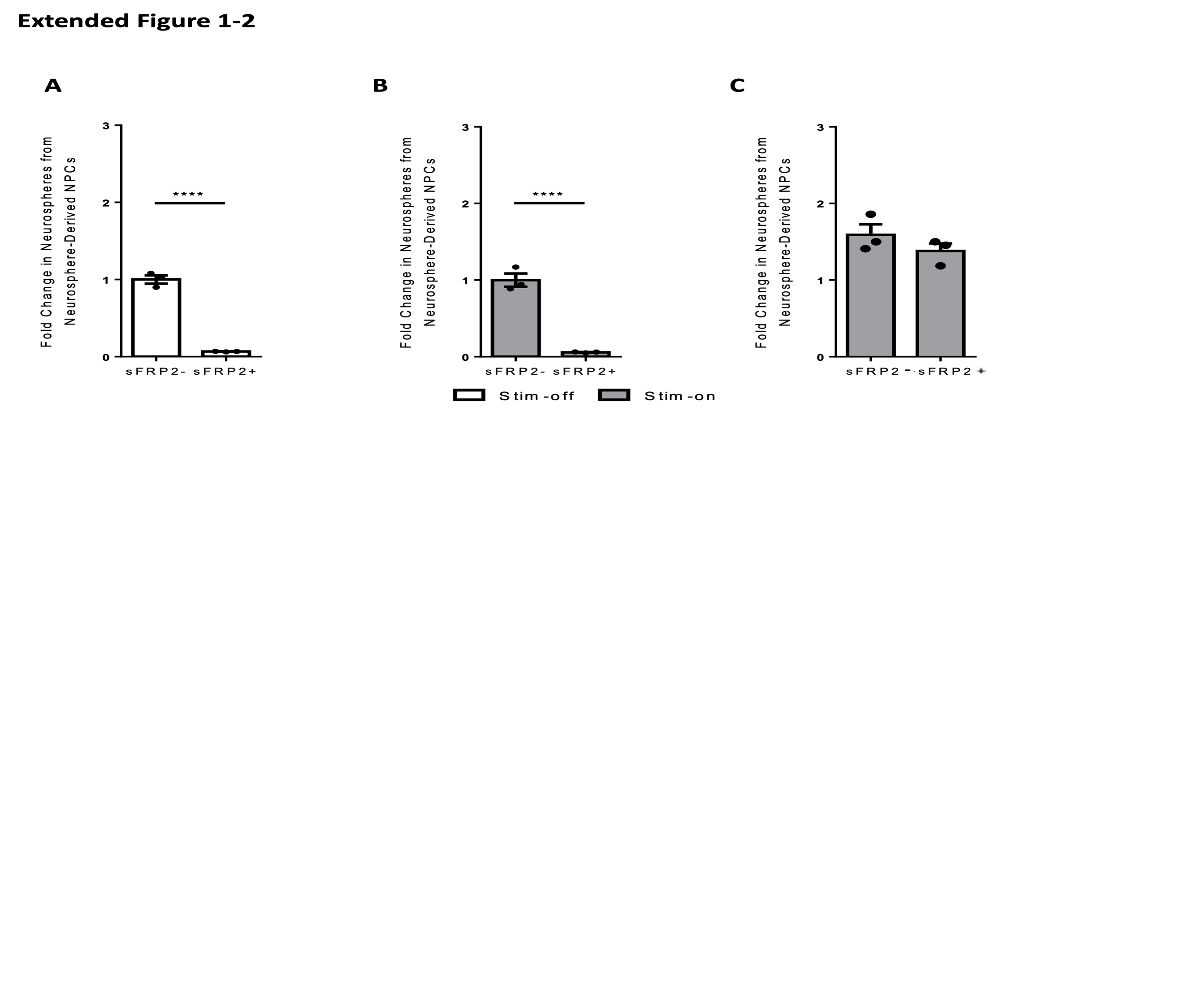

Supplement: Extended Data Figure 1-2 — Comparison of fold changes in neurospheres following stimulation with and without Wnt inhibitor (sFRP2). A, sFRP2 blocked the vast majority of neurosphere growth in the stim-off cultures as predicted with a 16-fold decrease of stem cells and (B) a 17-fold decrease in stim-on conditions (****p < 0.0001). C, Blocking symmetric division does not inhibit the increase in neurospheres following electrical stimulation; n = 3 independent experiments, two-tailed unpaired t test, p = 0.3. Download Figure 1-2, TIF file. [file enu-eN-NWR-0273-20-s05.tif]
