# Supplementary material for: Electric Field Application In Vivo Regulates Neural Precursor Cell Behavior in the Adult Mammalian Forebrain
Source: eNeuro. 2020 Aug 21;7(4):ENEURO.0273-20.2020. doi: 10.1523/ENEURO.0273-20.2020 (PMC7452733; doi:10.1523/ENEURO.0273-20.2020)
Supplement: Extended Data Figure 3-2 — Neurosphere counts from in vivo cortical stimulation. Data reported in mean ± SEM; n = 3 mice per group. Download Figure 3-2, DOC file. [file enu-eN-NWR-0273-20-s03.doc]

**Figure 3-2: Neurosphere counts from *in vivo* cortical stimulation**

| **Condition** | **Stim-off**  **(spheres/5,000 cells)** | **Stim-on**  **(spheres/5,000 cells)** |
| --- | --- | --- |
| 1 Day Post-Stimulation Contralateral | 4.6±0.3 | 5.6±0.4 |
| 1 Day Post-Stimulation Ipsilateral | 4.2±0.2 | 10.4±1.4 |
| 3 Days Post-Stimulation Contralateral | 10.4±0.5 | 10.1±0.4 |
| 3 Days Post-Stimulation Ipsilateral | 11.34±1.1 | 17.4±0.6 |

Data are reported as mean ± SEM.
