# Supplementary material for: Electric Field Application In Vivo Regulates Neural Precursor Cell Behavior in the Adult Mammalian Forebrain
Source: eNeuro. 2020 Aug 21;7(4):ENEURO.0273-20.2020. doi: 10.1523/ENEURO.0273-20.2020 (PMC7452733; doi:10.1523/ENEURO.0273-20.2020)
Supplement: Extended Data Figure 4-1 — Neurosphere counts from in vivo striatal stimulation. Data reported in mean ± SEM; n = 3 mice per group. Download Figure 4-1, DOC file. [file enu-eN-NWR-0273-20-s04.doc]

**Figure 4-1: Neurosphere counts from *in vivo* insulated and striatal stimulation**

| **Condition** | **Stim-off**  **(spheres/5,000 cells)** | **Stim-on**  **(spheres/5,000 cells)** |
| --- | --- | --- |
| Insulated Stimulation Contralateral | 4.0±0.2/5,000 cells | 5.0±0.3/5,000 cells |
| Insulated Stimulation Ipsilateral | 4.3±0.2/5,000 cells | 8.0±0.1/5,000 cells |
| Striatal Stimulation Contralateral | 4.1±0.1/5,000 cells | 4.7±0.1/5,000 cells |
| Striatal Stimulation Ipsilateral | 5.1±0.1/5,000 cells | 14.6±0.3/5,000 cells |

Data are reported as mean ± SEM.
